# Exploring Sprint Kinematics in American Football Athletes After Anterior Cruciate Ligament Reconstruction

**DOI:** 10.1111/sms.70200

**Published:** 2026-01-11

**Authors:** Naoaki Ito, Yi‐Chung Lin, Jack A. Martin, Silvia S. Blemker, David A. Opar, Stephanie A. Kliethermes, Bryan C. Heiderscheit, HAMIR Consortium Investigators, A. Wayne Johnson, Anthony Beutler, Anthony Nguyen, Ashleigh Homer, Brett Mortensen, Casey Metoyer, Claire Tanaka, Dain Allred, Danielle Heidt, Darren Campbell, Emma Remington, Erin Hammer, Geoffrey Baer, Jack Hickey, Jennifer Sanfilippo, John Wagle, Jonathon Hauenstein, Jordan Reyes, Josh Huff, Kenneth Lee, Kurrel Fabian, Lara Riem, Malorie Wilwand, Matthew Cousins, Matthew Kuehl, Michael Moll, Mikel Joachim, Nicholas Port, Nirav Maniar, Olivia DuCharme, Ryan Peot, Ryan Timmins, Sarah Sund, Xue Feng

**Affiliations:** ^1^ Department of Orthopedics and Rehabilitation University of Wisconsin – Madison Madison Wisconsin USA; ^2^ Badger Athletic Performance Program University of Wisconsin – Madison Madison Wisconsin USA; ^3^ Sports Performance, Recovery, Injury and New Technologies (SPRINT) Research Centre Australian Catholic University Melbourne Australia; ^4^ School of Behavioral and Health Sciences Australian Catholic University Melbourne Australia; ^5^ Department of Mechanical Engineering University of Wisconsin – Madison Madison Wisconsin USA; ^6^ Springbok Analytics Charlottesville USA; ^7^ Department of Biomedical Engineering University of Virginia Charlottesville Virginia USA

**Keywords:** biomechanics, gait, inertial measurement unit, knee, orthopedics, running, sports medicine

## Abstract

The purpose of this study was to compare stance time and knee joint kinematics between limbs during sprinting in Division‐1 collegiate American football athletes with a history of anterior cruciate ligament reconstruction (ACLR). This secondary analysis used data from an ongoing multicenter prospective cohort study of NCAA Division‐1 American football athletes. Sprint biomechanics were collected using wearable inertial measurement units during on‐field, maximal effort overground sprints. Knee kinematic variables of interest included stance time, knee flexion angle at initial contact, peak knee flexion angle, and knee flexion excursion during the stance phase from 2 to 5 strides near peak sprinting speed. Linear mixed effects models were used to evaluate the main effects of limb (involved vs. uninvolved), time from surgery, and their interaction on variables of interest, adjusted for peak sprint speed. Twenty male athletes (mean age: 21.2 ± 1.3 years; time from surgery: 28.0 ± 18.5 months) were fully participating in American football without limitations at the time of testing and met inclusion criteria. No significant limb × time interactions (*p* = 0.3–0.60) or main effects of limb (*p* = 0.23–0.84) or time (*p* = 0.08–0.84) were observed for any of our variables of interest. Division‐1 American football athletes approximately 2 years post‐ACLR did not demonstrate asymmetrical knee joint kinematics or stance time during sprinting. While gait asymmetries are commonly observed after ACLR during submaximal walking and running, these differences may diminish at high effort levels required during sprinting. IMU‐based methods provide a clinically feasible approach for assessing on‐field sprint biomechanics in athletes after ACLR.

## 1 Introduction

Aberrant walking and running biomechanics such as shorter stance times (antalgic gait) and lower knee flexion angles during weight acceptance are commonly seen in patients acutely and potentially for years after anterior cruciate ligament reconstruction (ACLR) surgery [1, 2, 3, 4]. Movement asymmetries during gait after ACLR are associated with poor outcomes such as the development of post‐traumatic knee osteoarthritis or suboptimal athletic performance [4, 5, 6, 7, 8]. Sprinting is a crucial part of performance in sports such as American football, where the prevalence of ACL injuries is high [9]. To date, however, little is known about the impact of ACL injuries and subsequent ACLR on sprint biomechanics, as well as the clinical and performance‐based implications of asymmetrical sprinting. Specifically, to our knowledge, knee kinematics during sprinting have never been studied in athletes after ACLR, and it is unknown whether the expected kinematic deficits we have observed in sub‐maximal running [10] are also present during sprinting.

Biomechanics during sprinting (greater than 80% effort) has been seldom studied in this population due to challenges such as movement artifacts from motion capture markers, spatial constraints of traditional 3D motion capture systems, and limitations of treadmill testing at high speeds. While some studies have successfully used optical 3D motion capture during sprinting in other populations [11, 12], the equipment is often specialized, expensive, and requires large spaces to allow athletes to reach near top speeds. Furthermore, the reproducibility and feasibility of longitudinal testing become more challenging as methodologies increase in complexity and require more equipment and time to complete. Wearable IMUs address these limitations by combining accelerometers, gyroscopes, and magnetometers in portable devices that eliminate spatial constraints, making feasible and repeatable sprint kinematic assessment possible [13, 14].

The ongoing *Hamstring Injury (HAMIR)* study (ClinicalTrials.gov ID: NCT05343052) [15], a multi‐center prospective cohort study, has successfully collected on‐field sprint biomechanics data from National Collegiate Athletic Association (NCAA) Division 1 American football athletes between the 2022–2024 football seasons using established IMU methods [13]. The purpose of this study was to compare stance time and knee joint kinematics between limbs during sprinting in a subset of this cohort with a history of ACLR. We hypothesized that shorter stance times and lower knee flexion angles during the stance phase would be observed in the involved limb compared to the uninvolved limb in athletes after unilateral ACLR.

## 2 Materials and Methods

Data from an ongoing multicenter prospective cohort study with NCAA Division‐1 collegiate American football athletes (ClinicalTrials.gov ID: NCT05343052) [15] was used for analysis. From all athletes enrolled in the parent study, those who had a history of primary unilateral ACLR, were fully participating in American football related activities without restriction, and completed subsequent sprint biomechanics testing as part of the parent study were included. Surgical history and graft type were self‐reported by each athlete, with the medical team at each participating university verifying the information for accuracy when possible. All participants in the study were informed of the potential risks and benefits associated with the parent study and provided written consent through an IRB‐approved process (IRB ID: 202–1420).

### 2.1 Sprint Biomechanics Testing

All athletes performed sprint testing on turf fields in cleats while wearing seven IMUs (Xsens MVN Awinda system, Movella, Enschede, The Netherlands): one on the sacrum and three on each lower limb (thigh, shank, and foot) according to the Xsens Awinda manual [16]. Each IMU contained a triaxial accelerometer (±160 m/s^2^), gyroscope (±2000°/s), and magnetometer (±1.9 G). Details of the Xsens Awinda system are available from the whitepaper by Paulich et al. [17]. Kinematic data were recorded at 100 Hz using the IMU system throughout each sprint trial. Prior to data collection, participants underwent the standard N‐pose calibration procedure per manufacturer recommendations using MVN Analyze software. The calibration protocol incorporated each participant's height and foot length to estimate body dimensions. After completing a sufficient warm‐up, participants performed maximal effort overground sprints from a standing start. Sprint distances ranged from 20 to 60 yards (18.29–54.86 m), varying based on the position they played (i.e., athletes who played positions that commonly run longer distances ran further distances).

### 2.2 Data Processing

**FIGURE 1:**
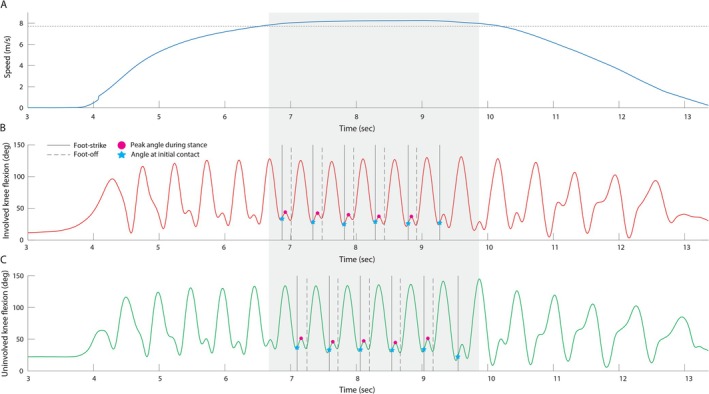
(A) Running speed, (B) involved limb knee flexion, and (C) uninvolved limb knee flexion measured from a representative athlete. The horizontal dotted line in A indicates the speed threshold used for analysis. Shaded areas indicate the region when running speed exceeds this threshold. Vertical solid and dashed lines mark foot‐strike and foot‐off events, respectively. Stars indicate initial contact events, and magenta circles denote instances of peak knee flexion during the stance phase.

A MATLAB (The Mathworks Inc., Natick, MA) script was developed to identify consecutive stride cycles during the steady‐state phase of running near peak speed. The selection process began with an initial speed threshold, derived either from the average speed measured by timing gates or set to a default value of 7 m/s when timing data was unavailable. The script then identified the running region where IMU‐captured speed exceeded this threshold value. Within this region, individual stride cycles were detected using peak knee flexion during the swing phase. For sprint trials covering distances greater than 30 yards (27.43 m), five consecutive strides per leg were extracted (Figure 1). For shorter sprint trials (less than or equal to 30 yards), two strides per leg were extracted due to the limited data collection window near peak speeds. If an insufficient number of strides were detected, the threshold was incrementally lowered by 0.1 m·s^−1^ until the required number of stride cycles was identified. Conversely, when additional strides were available, the region of interest was narrowed from both ends, prioritizing the central portion where speeds were most stable. Each stride cycle was defined from one foot contact to the subsequent foot contact of the same limb, and the stance phase was defined from foot contact to foot‐off. Foot‐ground contact events were initially identified using the proprietary detection algorithm within MVN Analyze [18]. To enhance consistency, these events were subsequently refined using another MATLAB script based on vertical acceleration data from the foot‐mounted IMU. The script employed a zero‐crossing technique to refine foot‐strike detection within a window defined by local negative and positive acceleration peaks, while foot‐off events were adjusted to align with local peak accelerations. This refinement process resulted in average timing adjustments of 0.01 s for foot‐strike and 0.02 s for foot‐off events.

Knee joint kinematic data during sprinting were analyzed to determine key biomechanical variables related to gait after ACLR [1, 2]. The joint angles obtained from each sprinting trial were processed using a fourth‐order Butterworth low‐pass filter with a cutoff frequency of 20 Hz. Four primary kinematic variables relevant to ACL injury and known to be altered in walking or sub‐maximal running gait were calculated and averaged across strides: stance time, knee flexion angle at initial contact (KFIC), peak knee flexion angle during the stance phase (PKF), and knee flexion excursion (calculated as the difference between the minimum knee flexion angle after initial contact and PKF). Our recent validation studies demonstrated that the present IMU‐based knee kinematics processing method achieves a root‐mean‐square‐error below 7° when compared to marker‐based motion capture during high‐speed treadmill running at 7.5 m/s [13, 19].

### 2.3 Statistics

Descriptive statistics for athlete demographics were calculated and reported. No a priori power analysis was performed as this was a secondary analysis from the parent perspective cohort study. Linear mixed effects models were created, and Type III analyses were performed [20] using Satterthwaite's method [21] to evaluate the fixed effects of limb (involved versus uninvolved), time from surgery (months), and the interaction effect (limb × time) on the kinematic variables of interest (α = 0.05). Time from surgery and the interaction effect were considered in the models since improvements in knee joint biomechanics are expected over time after ACLR [2]. The models were also adjusted for the fixed effects of peak running speed (m·s^−1^) which is known to impact stance time and joint kinematics during gait [22, 23, 24], and individual athletes were modeled using random intercepts. Estimated marginal means [25] with 95% confidence intervals were reported and interpreted for each variable of interest.

## 3 Results

Twenty male Division‐1 collegiate American football athletes met inclusion criteria (Time from surgery: mean ± standard deviation = 28.0 ± 18.5 months, median (range)) = 21.5 (7.3–68.1) months; age: 21.2 ± 1.3 years, height: 186.2 ± 8.1 cm, mass: 99.2 ± 15.8 kg, graft type: 1 (5%) quadriceps tendon autograft, 14 (70%) bone‐patellar tendon‐bone autograft, 5 (25%) hamstring autografts; American football position group [26]: 2 (10%) Bigs, 8 (40%) Power, 8 (40%) Skill, 2 (10%) Special. Average peak sprint speed was 7.5 ± 0.6 m·s^−1^, and athletes ran 39.8 ± 12.6 yards on average (7/20 athletes ran less than 30 yards).

### 3.1 Kinematic Differences Between Limbs During Sprinting

**FIGURE 2:**
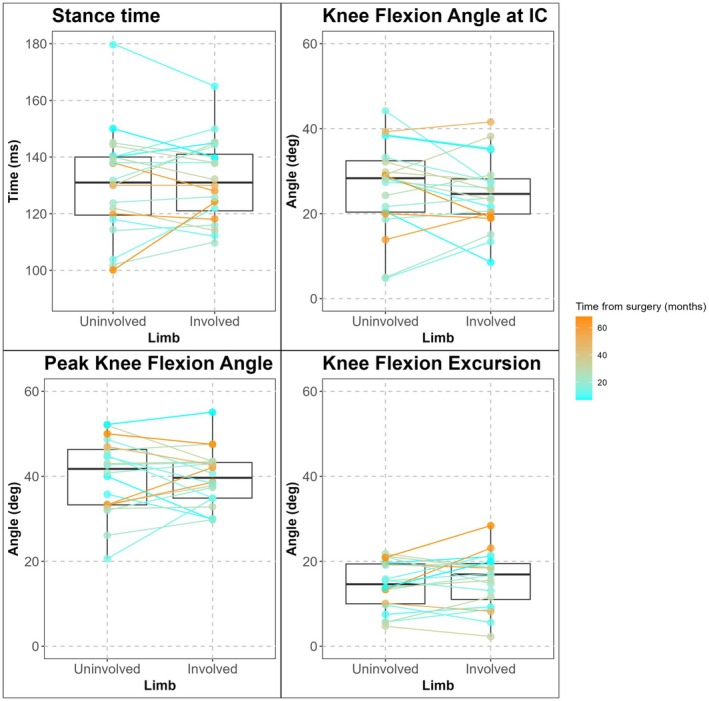
Boxplots for the involved and uninvolved limb biomechanical variables of interest. The color gradient represents the time from surgery in months.

No limb by time interaction effects (*p*‐values: 0.30–0.60), or main effects of limb (*p*‐values: 0.23–0.84) or time from surgery (*p*‐values: 0.08–0.84) were observed for any kinematic variables of interest (Tables 1 and 2, Figure 2). Between limb difference (uninvolved—involved, estimate [95% confidence interval]) for stance time was –1.3 [–6.2, 3.6] ms, knee flexion angle at initial contact was 1.4 [−2.0, 4.8], peak knee flexion angle was 0.3 [−2.8, 3.4], and knee flexion excursion was −1.2 [−3.2, 0.8] (Table 1).

**TABLE 1:** Main effects and estimates from a reduced model excluding non‐significant limb × time interaction effects. Interaction *p*‐values: Stance time = 0.60, Knee flexion angle at initial contact = 0.52, Peak knee flexion angle = 0.30, Knee flexion excursion = 0.35.

Fixed effects	Estimate	Lower 95% CI	Upper 95% CI	*p*
Model: Stance time (ms)				
Limb (Involved)	1.30	−3.29	5.89	0.59
Time from surgery (months)	−0.28	−0.58	0.02	0.08
Peak running speed (m/s)	−15.13	−24.24	−6.03	< 0.01
Model: Knee flexion angle at initial contact (deg)				
Limb (Involved)	−1.42	−4.60	1.75	0.39
Time from surgery (months)	−0.02	−0.25	0.20	0.84
Peak running speed (m/s)	−1.56	−8.37	5.26	0.66
Model: Peak knee flexion angle (deg)				
Limb (Involved)	−0.29	−3.18	2.59	0.84
Time from surgery (months)	0.05	−0.12	0.22	0.57
Peak running speed (m/s)	2.98	−2.23	8.19	0.28
Model: Knee flexion excursion (deg)				
Limb (Involved)	1.21	−0.69	3.10	0.23
Time from surgery (months)	0.08	−0.05	0.20	0.24
Peak running speed (m/s)	3.88	0.07	7.68	0.06

**TABLE 2:** Estimated marginal means [95% Confidence Intervals] for the four biomechanical variables of interest and between limb differences.

Variables	Uninvolved limb	Involved limb	Between limb difference
Stance time (ms)	130.5 [124.3, 136.8]	131.8 [125.6, 138.1]	−1.3 [−6.2, 3.6]
Knee Flexion Angle at Initial Contact (deg)	26.3 [21.7, 31.0]	24.9 [20.3, 29.5]	1.4 [−2.0, 4.8]
Peak Knee Flexion Angle (deg)	39.9 [36.3, 43.5]	39.6 [36.0, 43.3]	0.3 [−2.8, 3.4]
Knee Flexion Excursion (deg)	14.4 [11.8, 17.0]	15.6 [13.0, 18.2]	−1.2 [−3.2, 0.8]

## 4 Discussion

The primary finding from this study was that Division‐1 collegiate American football athletes who had fully returned to sport after ACLR may not present with asymmetrical stance time and stance phase knee kinematics during maximal effort on‐field sprinting. While asymmetrical gait mechanics are known to persist during walking and sub‐maximal effort running after ACLR, this same trend was not observed with sprinting in our cohort. To our knowledge, this is also the first study to investigate on‐field sprint kinematics in elite athletes after ACLR, adding to the growing body of literature that is beginning to implement wearable technology to assess biomechanics during various relevant movements on‐field [27]. We believe this analysis provides clinicians and researchers with a clinically feasible approach to collecting biomechanical data and reference values for knee kinematics in athletes after ACLR during overground sprinting.

Unlike treadmill walking and running gait [3, 4, 28], knee kinematics during overground sprinting were not found to be asymmetrical after ACLR in our cohort, even after accounting for time since surgery. Several factors may explain these findings. Most interestingly, we may not have observed knee kinematic asymmetries because sprinting, unlike running at sub‐maximal speeds, requires the athlete to use the body's maximum capacity to perform. Literature has shown that during sub‐maximal running, peak knee flexion angle asymmetries during stance reduce as running speed (i.e., effort) increases due to the involved limb undergoing proportionately more flexion with faster speeds compared to the uninvolved limb [23]. This phenomenon can mainly be attributed to quadriceps dysfunction and subsequent disuse strategies during gait commonly observed after ACL injury [29, 30, 31, 32]. When athletes walk or run at low effort levels, they are still capable of sufficiently completing the locomotive task while minimizing use of the knee joint and relying on compensatory mechanics. During high effort level sprints, however, the body may be forced to use the full capacity of the knee joint. Our findings from sprinting may be an extension of this previous knowledge from slower speed running, where knee flexion angle asymmetries that may be present during lower effort level gait are reduced to a minimum or do not exist due to the high demands of the task. Additional parameters of lower extremity biomechanics including kinetic metrics (i.e., joint moments and power), however, must also be considered in future studies to make a more concrete statement on this theory. While lack of kinematic asymmetry was not seen in our cohort, underlying alterations to sprint kinetics from within limb (from the hip and ankle joints) and between limb (from the uninvolved limb) compensations or overall differences with healthy controls may still be present.

Our findings could also be explained by the homogeneous cohort of elite athletes in Division‐1 collegiate American football that we studied. With higher baseline athletic abilities, greater access to resources, and dedicated time for recovery compared to recreational athletes, those in this study may have recovered more effectively than other cohorts studied in previous literature [3]. They may have also undergone more stringent return‐to‐sport clearance criteria (i.e., requiring them to clear higher thresholds on functional tests compared to the recreational athlete population prior to performing on‐field) [33, 34] to continue competing at an elite level. Additionally, those who were unable to restore their knee function may not have returned to sport at the same level and hence were not included in our analysis leading to the current findings. It is important to note, however, that recent work in Division‐1 athletes, including American Football athletes, demonstrated that athletes continue to run (not sprint) with asymmetrical knee kinetics and kinematics up to 1 year after ACLR [24].

Despite the similar sprinting mechanics between limbs, our previous analysis from the same parent study found that quadriceps atrophy of the involved limb persisted among athletes who underwent a prior unilateral ACLR and were now competing without restrictions [35]. While raw muscle volume, strength, and biomechanics should be considered as independent domains and targets for rehabilitation, the connections between structure, strength and function cannot be ignored [28, 31, 36, 37, 38]. We must acknowledge the continued difficulty athletes face in restoring quadriceps function after ACL injury, and while sprint kinematic asymmetries may not have been present in this cohort, emphasis should continue to be placed on restoring quadriceps and overall knee extension function after ACL injury to aid in the recovery of gait and optimizing performance.

Readers may also begin to consider the kinematic data presented in this study as a reference for future research and clinical implementation of IMU‐based sprint kinematic testing for athletes after ACLR. The methods used in this study have been validated against 3D optical motion capture during maximal speed sprinting on a non‐motorized treadmill [13]. While there is no direct validation of our approach against overground sprinting, the knee flexion angles and excursions (Table 1) we observed during the stance phase are comparable with other work that has quantified overground sprint knee kinematics (e.g., knee flexion angle at initial contact = 26.3 ± 4.7°) during low acceleration conditions (i.e., near maximum speeds) in healthy individuals using 3D motion capture [11].

There are limitations to acknowledge in this study. This study was limited to studying stance phase kinematics, but underlying kinetic asymmetries, such as knee joint moment or power, could be present during sprinting. Further studies incorporating ground reaction force data to perform inverse dynamics may be necessary to answer the impact of ACLR on on‐field sprint kinetics. Advancements in markerless motion capture technology [39, 40] and integration with built‐in on‐field forceplates may allow for this. Such methods, however, sacrifice clinical feasibility compared to IMU‐based methods due to the extensive setup. Alterations to other joints, such as the hip and ankle joint or movements in the frontal or transverse plane were not explored in this study. The described IMU‐based methods are most valid compared to traditional optical motion capture methods in the sagittal plane [13, 19] and most deficits are known to occur in the sagittal plane after ACL injury. Future studies may begin to explore biomechanics in other planes and joints as methods continue to improve. The cohort we tested was also limited in sample size, had a wide spread in time from surgery, and the distances they sprinted varied based on the position played. It is possible that those who ran shorter distances may not have reached peak speeds; hence, kinematics during the acceleration phase of the sprints were captured. Longitudinal testing after ACLR in a larger cohort with consistent methodology may allow for further investigating the effects of ACLR on sprint biomechanics. Other variables relevant to recovery from ACLR such as time since return to play from ACLR, quadriceps strength, concomitant injuries, graft type were not considered in this analysis due to the small sample size and limited information collected specific to ACL injury since it was not the primary aim of the parent study. Findings from this study may also not be generalizable to other athletes from demographics outside of high‐level American football players. The data we present, however, serves as an excellent reference for those who work with this population, and while translation of overall findings can be expected beyond American football players, further testing is also warranted in other populations such as female athletes.

## 5 Perspective

Knee kinematic and stance time asymmetries were not present during on‐field sprinting in a cohort of Division 1 collegiate American football athletes after unilateral ACLR. Unlike asymmetries that are observed for years after ACLR during submaximal effort gait such as walking and running, maximal effort sprinting may require the use of the knee's maximum capacity to perform, possibly explaining our findings. This study is the first of its kind to successfully measure knee joint kinematics during on‐field sprinting in a cohort of elite athletes after ACLR. Future studies may implement this clinically feasible approach to continue studying sprint biomechanics after ACL injuries.

## Funding

This work was supported by the National Football League Foundation.

## Conflicts of Interest

The authors declare no conflicts of interest.

## Data Availability

The data that support the findings of this study are available on request from the corresponding author. The data are not publicly available due to privacy or ethical restrictions.
